# Validity and usefulness of members reports of implementation progress in a quality improvement initiative: findings from the Team Check-up Tool (TCT)

**DOI:** 10.1186/1748-5908-6-115

**Published:** 2011-10-03

**Authors:** Kitty S Chan, Yea-Jen Hsu, Lisa H Lubomski, Jill A Marsteller

**Affiliations:** 1 Department of Health Policy and Management, Johns Hopkins Bloomberg School of Public Health, 624 North Broadway, Baltimore, MD 21205, USA; 2 Department of Anesthesiology and Critical Care Medicine, Johns Hopkins School of Medicine, 1909 Thames Street, Baltimore, MD 21231, USA

## Abstract

**Background:**

Team-based interventions are effective for improving safety and quality of healthcare. However, contextual factors, such as team functioning, leadership, and organizational support, can vary significantly across teams and affect the level of implementation success. Yet, the science for measuring context is immature. The goal of this study is to validate measures from a short instrument tailored to track dynamic context and progress for a team-based quality improvement (QI) intervention.

**Methods:**

Design: Secondary cross-sectional and longitudinal analysis of data from a clustered randomized controlled trial (RCT) of a team-based quality improvement intervention to reduce central line-associated bloodstream infection (CLABSI) rates in intensive care units (ICUs).

Setting: Forty-six ICUs located within 35 faith-based, not-for-profit community hospitals across 12 states in the U.S.

Population: Team members participating in an ICU-based QI intervention.

Measures: The primary measure is the Team Check-up Tool (TCT), an original instrument that assesses context and progress of a team-based QI intervention. The TCT is administered monthly. Validation measures include CLABSI rate, Team Functioning Survey (TFS) and Practice Environment Scale (PES) from the Nursing Work Index.

Analysis: Temporal stability, responsiveness and validity of the TCT.

**Results:**

We found evidence supporting the temporal stability, construct validity, and responsiveness of TCT measures of intervention activities, perceived group-level behaviors, and barriers to team progress.

**Conclusions:**

The TCT demonstrates good measurement reliability, validity, and responsiveness. By having more validated measures on implementation context, researchers can more readily conduct rigorous studies to identify contextual variables linked to key intervention and patient outcomes and strengthen the evidence base on successful spread of efficacious team-based interventions. QI teams participating in an intervention should also find data from a validated tool useful for identifying opportunities to improve their own implementation.

## Background

Team-based interventions are effective for improving safety and quality of healthcare for a variety of settings and patient populations [1]. In fact, substantial reductions in central line-associated bloodstream infection (CLABSI) rates for intensive care units (ICUs), shorter hospital stays for stroke patients, and improvements in end-of-life care have been reported for team-based interventions [2-4]. However, significant variation across teams in the achievement of desired outcomes has also been observed, even within successful quality improvement (QI) initiatives or collaboratives (*e.g*., [5]). For example, Mills and Weeks reported that the proportion of successful teams ranged between 51% and 68% for collaboratives focused on adverse drug events, improving safety in high risk areas, home-based primary care for dementia patients, reducing falls and injuries due to falls, and improving compensation and pension examination processes [6]. Similarly, Lynn *et al*. reported that 27% and 47% of the teams in two collaboratives on end-of-life care achieved substantial improvements in outcomes, even though 85% of the teams reported making key changes to their systems to improve care [2]. Finally, Schouten *et al*. found that the average length of stay varied substantially across teams, although the collaborative realized an overall reduction of five days from the hospital stay of stroke patients [4].

In these types of interventions, contextual factors, such as team characteristics and organizational support, significantly affect the level of implementation success. In their analysis of the factors contributing to successful collaboratives, Øvretveit *et al*. highlighted the role of effective team functioning, communication, and relationships for successful collaboratives [5]. Lemieux-Charles and McGuire noted in their review that high-functioning teams have positive communication patterns, low levels of interpersonal conflict, and high levels of collaboration, coordination, cooperation, and participation [1]. Furthermore, these processes are positively associated with perceived team effectiveness. Greater team effectiveness can lead to stronger intervention effects and more positive outcomes. Shortell *et al*. reported that greater perceived team effectiveness was associated with a larger number of and deeper changes being made by teams participating in collaboratives to improve care for the chronically ill [7]. Schouten *et al*. found that better team functioning was associated with shorter length of stay and better adherence to recommended stroke care [4]. In fact, QI team characteristics explained 40% of the variance in length of hospital stay and 53% of the variance in adherence to recommended stroke care.

In addition to teamwork, leadership support and available resources may be important context variables. However, team functioning, leadership and organizational support can vary across teams and, notably, change over the course of an intervention [6]. Monitoring implementation context can help teams and QI collaborative faculty and leadership in addressing problems that hinder progress. Furthermore, identifying factors that support successful implementation can help ensure that positive outcomes are achieved when interventions spread to other settings.

Despite the importance of measuring context, the science of what domains to measure and how to measure them remains immature. Qualitative reports of team activities and perceptions have been used to study implementation processes in QI collaboratives [8-10]. However, these methods can be burdensome to use on a routine basis. Validated measures such as the 38-item Team Climate Inventory [11] assessing workgroup innovation and organizational climate are available, but may not be tailored to the team processes or implementation concerns of a particular intervention. Given that data collection is one of the major challenges faced by teams participating in collaboratives [5], having a measure that is relevant, evaluates multiple domains, and is feasible to administer on a routine basis is necessary for successfully monitoring progress for a given intervention.

The goal of this study is to demonstrate that a short instrument, the Team Check-up Tool (TCT), can provide reliable and valid contextual data for monitoring team progress within a QI intervention. This instrument and an earlier version have been used to monitor team progress and implementation context for large-scale QI interventions to reduce bloodstream infections in the ICU [12,13]. Evidence of temporal reliability, responsiveness and construct validity of the TCT will support its future use as the intervention spreads to additional hospitals and other settings. Finally, the TCT can serve as a model for developing comparable measures for other team-based QI interventions.

## Methods

### Data source

Data for this study were drawn from a multi-centered clustered randomized controlled trial (RCT) of a team-based QI intervention conducted in 46 ICUs [13]. The ICUs were located within 35 faith-based, not-for-profit community hospitals across 12 states. These hospitals are part of two Adventist health systems. QI teams were comprised of nurses and physicians from each participating ICU, and included senior executives from hospital administration. A nurse manager from the unit, a nurse educator, or an infection preventionist typically served as the team leader. The team is expected to implement the intervention and educate other clinical staff within the ICU in the targeted safety practices. Team members completed monthly TCTs. CLABSI data were obtained monthly from the infection preventionist at each hospital. Practice Environment Scale-Nursing Work Index (PES-NWI) and Team Functioning Survey (TFS) data, each collected once during the study period, are used to validate the TCT measures. Study measures [7,12,14] are described in greater detail below.

The intervention was a phased RCT, with 22 ICUs (intervention group II) randomized to begin the intervention seven months after the 23 ICUs in intervention group I initiated the intervention. Another ICU joined the project after the randomization process had completed and participated in intervention group II. Overall, intervention-I group contributed 19 months of data, while intervention-II group contributed 12 months of data. The additional seven months of data from intervention-I group provided a longer longitudinal assessment of the measure and therefore were retained in the analysis. Details regarding randomization and other aspects of the parent study are provided elsewhere [13].

### Primary measures

**Figure 1:**
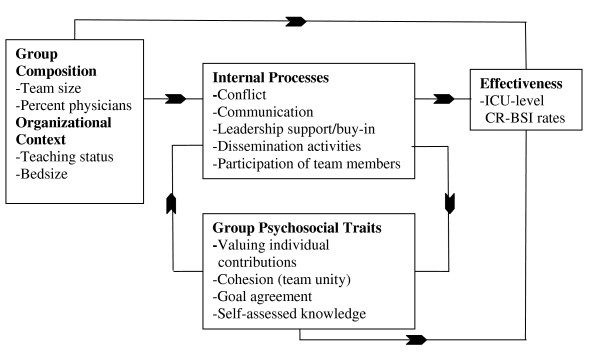
**Conceptual Framework underlying the Team Check-up Tool**.

The Team Check-up Tool (TCT) is an original instrument that assesses the following aspects of a QI intervention: intervention activities; perceived unit-level intervention-related behavior; implementation processes and context such as leadership support and available resources; and perceived barriers to team progress. The TCT was developed by the Johns Hopkins Quality and Safety Research Group (QSRG) for use in the Keystone ICU project [12] and was later modified for use in the project described here. It is a brief tool suitable for routine completion over the course of an intervention to assess the progress of a specific team-based QI intervention. Each month, ICU QI team leaders collected the TCT from team members and mailed them to the QSRG using a paper form provided by the research team. QI team leaders were asked to provide confidentiality for team members by collecting the surveys folded and placing them in an envelope without reviewing them. The research team provided technical support for data collection through conference calls and meetings, but no financial incentive was provided for filling out the tool. In general, participants estimated that it took seven to ten minutes to complete the TCT. We focus on the reliability and validity of intervention activities, perceptions of unit-level behavior, and barriers to team progress. We did not examine items that were expected to vary significantly from month to month and for which data were not available to validate these reports. These items include queries on the number of times the team met with each other, the senior leadership or the board at the hospital, staff turnover, and distracting events. The conceptual framework for the TCT is presented in Figure 1 and a copy of the TCT is provided in Additional file 1, Table S1.

### Intervention activities

The intervention was developed by the QSRG. The Comprehensive Unit-based Safety Program (CUSP) as used in this collaborative was a five-step process intended to improve safety, teamwork, and communication [15]. Activities included: morning briefing, executive partnership, shadowing, daily goals, learning from a defect, and a Science of Safety video. Educational activities provided to unit staff may have included: internal seminar, infectious control visit/talk, in-services/demo, new written policy, posted steps, and putting protocol on clipboards. Each team may participate in one or more of these activities in any given month. Further details on the intervention, and the suggested implementation framework (known as the '4 Es') have been published elsewhere [3,16-18]. We calculated a sum of CUSP activities and a sum of educational activities to reflect two aspects of the intensity of intervention activity.

### Perceived unit-level intervention-related behavior

QI team members were asked to report their perception of the proportion (*i.e*., few, some, most, all) of unit staff that consistently used the five behaviors that the study intervention sought to increase: appropriate hand hygiene; chlorhexidine skin preparation; full barrier precautions during line insertion; subclavian vein placement; and ask daily about removing unnecessary lines. We examined these items individually and as a sum across the five behaviors. Unit-level performance for a behavior was indicated if the member reported most or all of unit staff consistently performed the activity. A summed score was then calculated as the number of the five behaviors performed by the unit.

### Barriers to team progress

Team members were asked to indicate the frequency (*i.e*., never/rarely, under one-half the time, one-half the time, over one-half the time, almost always/always) with which thirteen potential barriers slowed team progress. These barriers include: insufficient knowledge of evidence base for intervention, low consensus within team regarding goals, lack of time, lack of QI skills, lack of buy-in from other staff on the unit, data collection burden, lack of leadership support, insufficient autonomy or authority, and inability of team to work together. We examined these items individually and as a summed score. The summed score was calculated by adding the number of individual barriers that were each faced one-half the time or more. The summed score has a range of 0 to 13. There were also five items (questions 15 m1 to 15m5, see Additional file 1, Table S1) on contributors to poor team function that participants were asked to respond to if they indicated the team could not work together more than one-half the time (question 15 m). These included: insufficient participation by one or more members; some members do not value contribution of others; low or no feeling of being a team; personality conflicts; and poor conflict resolution skills. Since only team members who reported poor team functioning responded to these five questions, they were not included in the summed score or further evaluated due to insufficient number of responses.

Responses for the TCT are analyzed at the individual level and at the ICU level in our study. For ICU-level analyses, team member reports, if there are more than one, are averaged across individual team member reports for barrier items to obtain a group-level value for the ICU each month.

### Validation measures

#### Practice environment scale (PES)

The PES is part of the Nursing Work Index (NWI) that was designed to measure organizational factors associated with job satisfaction and the quality of nursing care delivery [14]. The PES measures five components of hospital culture: nursing participation in hospital affairs; nursing foundations for quality of care; nurse manager ability, leadership, and support of nurses; staffing and resource adequacy; and the degree of collegial nurse/physician relationships. The five subscales have been validated through a confirmatory factor analysis and Cronbach's alpha reliability estimates range between 0.71 and 0.84 [14]. The PES-NWI was filled out at baseline by all nurses working in the participating ICUs. Data from the baseline administration were used to assess cross-sectional discriminant validity with the TCT barriers to team progress measures. Data for the TCT was the average score of the first quarter (March through May 2007). Only ICUs in the first intervention group were included because the second intervention group submitted their first TCT seven months later when they began implementing the intervention.

#### Team functioning survey (TFS)

The TFS [7] is adapted from the team effectiveness instrument originally developed by G. Ross Baker and colleagues at the University of Toronto, and modified for use in the Improving Chronic Illness Care Evaluation http://www.rand.org/health/projects/icice.html. Respondents agreed or disagreed, on a scale from 1 to 7, with statements of how the team worked together and its environment. There are five subscales for this instrument: information/help available; organizational support; team self-assessed skill; participation and goal agreement; and team autonomy. This measure has been shown to have good internal consistency, with Cronbach's alpha for the five subscales ranging from 0.85 to 0.95 [7]. Overall perceived effectiveness was positively related to both the number and depth of changes made to improve care for the chronically ill. This instrument was administered at the end of the intervention period (August through October 2008). The TCT data used in the TFS analyses was the average score of the last quarter (July through September 2008).

#### CLABSI (central line-associated bloodstream infection)

The number of CLABSIs occurring within an ICU and number of catheter-line days were collected monthly by the hospital infection preventionist using the Centers for Disease Control and Prevention's (CDC) definitions and standards http://www.cdc.gov. These data were reported via the hospital system's corporate headquarters. Primary CLABSIs were determined using the following criteria: bloodstream infections in ICU patients aged 18 years and older with a laboratory confirmed CLABSI who had central lines in place within the 48-hour period before the development of the infection. Non-ICU patients, patients without central lines, secondary bloodstream infections, and those present or incubating within 72 hours of admission to the unit were excluded. The rate of CLABSI is calculated by dividing the number of infections by the number of catheter-line days and is commonly expressed as the number of CLABSI per 1000 line days.

### Analysis

#### Measure reliability

To examine temporal stability, we calculated average Spearman correlation (infection prevention behavior and team barrier items) and percent agreement (intervention CUSP, educational activities) during the third quarter of the intervention, when activities, infection prevention behaviors, and team progress barrier perceptions should be stable. We used percent agreement rather than kappa in our study due to the high expected agreement during the stable period. An ICU-level agreement statistic for each measure was determined by averaging the correlations or percent agreement between months seven and eight and between months eight and nine. Overall agreement for each measure was calculated by averaging across all ICUs that submitted enough TCTs for percent agreement calculation during this period (n = 31). We also reported internal consistency reliability, using Cronbach's alpha, for perceived group-level behavior and barriers to team progress. We did not calculate alpha for the CUSP and education activities. Given the nature of these activities, actively engaged teams may choose to undertake different activities during different intervention months. Therefore, in a particular month, participation in these activities may not be positively correlated. This violates a basic assumption underlying internal consistency that observed responses are driven by a latent unidimensional construct and therefore positively correlated.

#### Measure responsiveness

A measure that is expected to be used within QI initiatives must be able to reflect change when true change has occurred, while demonstrating stability when little real change has taken place. We examined measure responsiveness during a high activity period during early implementation and a low activity period later on when intervention implementation and team behavior are expected to have stabilized. For CUSP and educational activities, the first quarter is the high activity period and the third quarter is the low activity period. For each ICU, an ICU-level value is calculated that summarizes team member reports for each month. ICUs must have at least two months of TCT data within the quarter to be included (n = 31). As behavior is expected to lag intervention activities, the first two quarters are identified as the high activity period for infection prevention practices and team progress barriers. The third and fourth quarters are defined as the stable period. ICUs must have at least two TCTs in each quarter of the period to be included (n = 25). For intervention activities, we calculated the number of activities undertaken per month. For behavior and barrier measures, we calculated the average number of practices and barriers in each quarter. We used a paired t-test at the ICU level to determine if any of the changes, whether monthly or quarterly, were significantly different from zero.

To demonstrate the ability of the measure to track changes over time, we also graphed the bimonthly numbers of perceived infection prevention behaviors and the numbers of team progress barriers over the course of the intervention. Trends should reflect improved behaviors and lower barriers over time as ICU teams learn to work together and resolve differences within the team. All ICU-level data available for each month were used, with 41 ICUs contributing data for this analysis. For ICUs that had values for both months in a two-month period, the average of the two months was used. For those with only one month in a two-month period, we used the available value as an estimate for the average in the two-month period. As intervention group II, from the phased parent RCT, had data only up to month 12, the subsequent months include data only from the 23 ICUs in intervention group I. Correlation between these two measures over time was calculated.

### Measure validity

#### Construct validity

Construct validity is demonstrated when the measure under evaluation demonstrates associations that are expected for the underlying trait based on theory or prior empirical studies. We evaluated the construct validity of the intervention activities, unit infection prevention behavior, and perceptions of team progress barriers by examining their interrelationships. We hypothesized that greater concurrent CUSP and education activities would be associated with greater number of prevention behaviors undertaken by unit staff. Conversely, we hypothesized that a greater number of perceived barriers to team progress would be associated with lower numbers of prevention behaviors. Because these measures are all part of the TCT tool, we were able to perform these analyses using data from individual team member reports (n = 1,406).

#### Convergent and discriminant validity

Convergent validity is demonstrated when measures of similar constructs show significant associations with each other. We evaluated the convergent validity for the sum of team progress barrier items through Pearson correlation with the overall TFS score. Similarly, we examined the Pearson correlation of specific barrier items with related TFS subscales. Specifically, we expect: the barrier item on insufficient autonomy to be related to the TFS team autonomy subscale; the three leadership support barrier items to be related to the TFS organizational support subscale; and the barrier items on lack of team consensus and inability of team members to work together to be related to the TFS subscale of participation and goal agreement. Because higher scores indicate poorer team functioning for both the sum and individual barrier items, we expect to find significant negative correlations with the TFS. Twenty-two ICUs that submitted any TCT in the last quarter and also submitted the TFS were included in this analysis.

Discriminant validity is demonstrated by the lack of an association between measures of constructs that are expected to have little or no relationship with each other. The PES assesses an overall working environment that may have only distal, weak linkages to the dynamics within a specific QI team. Therefore, we hypothesize that we will find weak to no correlation of the sum of the barrier items with an overall score for the PES. Similarly, we hypothesize that individual barrier items will show weak correlation with the PES subscale on staffing and resource adequacy at the hospital level, which is expected to be weakly related to the barriers to team progress experienced within a small group intervention team. Fifteen ICUs in the intervention group I that submitted any TCT in the first quarter and also submitted the PES were included in this analysis. These analyses were performed at the ICU level because individual members cannot be linked between the TCT, TFS, and PES. Different representatives from the same ICU may have contributed reports for different measures.

#### Predictive Validity

Predictive validity is demonstrated when an important outcome or future event that is associated with the measured construct is observed empirically with the measure. We used the Cox proportional hazards model to examine predictive validity. We tested the association of the summed team progress barriers with: time to the first three months of no CLABSI, and time to first three months when five prevention behaviors were consistently performed by unit staff. Time was calculated in months. We hypothesized that teams with fewer reported barriers will achieve these desired outcomes in a shorter period of time. Twenty-two ICUs that submitted a TCT in the first month of the implementation were included in the CLABSI analysis. Fifteen ICUs that submitted a TCT in the first month and enough subsequent TCT reports to identify three consecutive months of unit prevention behaviors contributed to the infection prevention behavior analysis.

For item-level analyses, where *a priori *hypotheses were not proposed, we used the Bonferroni correction to account for multiple comparisons.

## Results

The ICUs included in this study come from hospitals located in 12 states, with representation from the western (CA, WA, OR), southern (FL, GA, KS, KY, NC, TN, TX) and mid-western continental states (IL) and Hawaii. Table 1 presents key characteristics of participating ICUs. Most of these ICUs were of mixed specialty, although 18% were coronary/cardiovascular ICUs. Among the 46 ICUs participating in the multicenter trial, an average of 51% submitted at least one TCT for each of the first 12 months of the intervention. Among those ICUs with at least one submitted TCT, the median number of TCT submitted by an ICU each month is 4.

**Table 1:** Characteristics of ICU sample

Description of ICUs	N = 46
No. of beds* (Mean, SD)	13 (7)
No. of nurses* (Mean, SD)	32 (19)
Type of ICUs*, %	
Medical	2
Surgical	2
Mixed	76
Neurosurgical	2
Coronary/Cardiovascular	18
**System, %**	
East	78
West	22
**Location, State, %**	
CA	15
FL	46
GA	4
HI	2
IL	13
KS	2
KY	2
NC	2
OR	2
TN	4
TX	4
WA	2
**Median number of TCT reports submitted, across all ICU-months**	4 (min: 1, max: 15)

* Data for these characteristics not available for 1 of the 46 ICUs included in our analyses.

### Measure reliability and responsiveness

#### Internal consistency

Cronbach's alpha was 0.78 for preventive behaviors and 0.91 for team barriers, indicating good reliability for both sets of items. As noted in methods, the assumption for alpha was not met for the CUSP and educational items and, therefore, not calculated.

#### Temporal stability

Temporal stability of individual items, assessed during a stable period in the third quarter, was good overall. Average monthly percent agreement ranges between 62% and 92% for individual CUSP activities and between 74% and 97% for educational activities. Average Spearman correlation for infection prevention behaviors, except for hand hygiene, is 0.58 to 0.71. The correlation for hand hygiene is -0.15. Further examination of the distribution of this item suggests that the low variance in this item may have contributed to this unexpected result. All the values for hand hygiene were between 3 and 4 for all three months, with most of the values between 3 and 3.5.

Among the perception of barrier items, the Spearman correlation ranges between 0.39 and 0.92, with 10 of the 13 items demonstrating at least moderate correlation (> 0.50) between month. The lack of data precluded calculation of average monthly correlation for the five items (questions 15 m1 to 15m5, see Additional file 1, Table S1) on contributors to poor team function. Participants were asked to respond to these questions only if they indicated the team could not work together more than one-half of the time. Consequently, only five to nine ICUs had any responses to these items and only one to four ICUs had consecutive data to allow agreement statistics to be calculated.

Evidence of temporal stability was also observed in the lack of significant change during the low activity period (Table 2).

**Table 2:** TCT responsiveness and temporal stability*

Change in TCT items and sum scores	High Activity (Change) Period	Low Activity (Stable) Period
Number of CUSP activities** (Range: 0 to 6)	**0.88 (p < 0.01)** **Monthly**, **1^st ^quarter**	**-0.08 (p = 0.70)** **Monthly**, **3^rd ^quarter**
Number of Educational activities** (Range 0 to 6)	**0.57 (p = 0.06)** **Monthly**, **1^st ^quarter**	**-0.28 (p = 0.15)** **Monthly**, **3^rd ^quarter**
Number of Infection Prevention Behaviors** (Range: 0 to 5)	**0.52 (p = 0.02)** **Quarterly**, **1**^**st **^**and 2**^**nd**^	0.01 **(p = 0.92)** **Quarterly**, **3**^**rd **^**and 4**^**th**^
Appropriate hand hygiene (Range: 1 to 4)	0.11 (p = 0.08)	-0.01 (p = 0.93)
Chlorhexidine skin preparation (Range: 1 to 4)	0.15 (p = 0.34)	-0.02 (p = 0.83)
Full-barrier precautions during line insertion (Range: 1 to 4)	0.22 (p = 0.04)	0.06 (p = 0.44)
Subclavian vein placement (Range: 1 to 4)	0.13 (p = 0.14)	0.04 (p = 0.73)
Removing unnecessary lines (Range: 1 to 4)	0.20 (p = 0.04)	0.03 (p = 0.73)
Number of Team Progress Barriers** (Range: 0 to 13)	**-0.62 (p = 0.18)** **Quarterly, 1**^**st **^**and 2**^**nd**^	**-0.36 (p = 0.33)** **Quarterly, 3**^**rd **^**and 4**^**th**^
Insufficient knowledge	-0.21 (p = 0.15)	-0.03 (p = 0.52)
Lack of team consensus	-0.28 (p = 0.15)	-0.25 (p = 0.13)
Not enough time	-0.17 (p = 0.40)	-0.01 (p = 0.94)
Lack of quality improvement skills	-0.32 (p = 0.11)	-0.11 (p = 0.16)
Not enough buy-in from other staff	-0.39 (p = 0.03)	-0.07 (p = 0.51)
Not enough buy-in from other physician staff	-0.35 (p = 0.02)	-0.06 (p = 0.78)
Not enough buy-in from other nursing staff	-0.33 (p = 0.11)	-0.04 (p = 0.63)
Burden of data collection	-0.29 (p = 0.22)	-0.11 (p = 0.36)
Not enough leadership support from executives	-0.15 (p = 0.23)	0.13 (p = 0.43)
Not enough leadership support from physicians	-0.21 (p = 0.17)	0.01 (p = 0.96)
Not enough leadership support from nurses	-0.27 (p = 0.01)	-0.01 (p = 0.94)
Insufficient autonomy/authority	-0.23 (p = 0.03)	-0.20 (p = 0.24)
Inability of team to work together	-0.04 (p = 0.47)	-0.04 (p = 0.60)

*Thirty-nine ICUs were included in the analysis (these 39 ICUs did not significantly differ from the seven ICUs excluded from the analyses in # beds, # MD intensivists, # nurses, type of ICUs, geographic region, nor time to first month of zero infections); Please refer to the Additional file 1, Table S1 for specific wording and response categories of each item: CUSP (item #1); educational activities (item #2); prevention behaviors (item #3a-e); barriers to team progress (item #15a to 15 m, 15 m1 to 15m5).

#### Measure responsiveness

In general, the measures of interest demonstrated good responsiveness, with score changes observed in the expected direction during the early period of implementation and more stable scores observed later on (see Table 2). Specifically, the number of intervention (CUSP and educational) activities increased significantly month to month in the first quarter and were smaller and not statistically different from zero in the third quarter.

Similarly, the perceived proportion of unit staff that consistently used infection prevention behaviors increased significantly early in the implementation stage (0.52, p = 0.02, from first to second quarter) then stabilized later in the implementation (0.01, p = 0.92, from third to fourth quarter). At the item level, the changes were largest for use of full barrier precautions (0.22, p = 0.04) and removing unnecessary lines (p = 0.20, p = 0.04).

The change in the sum of perceived barriers to team progress was in the expected direction, with greater decrease in barriers between the first two quarters than in the last two quarters. However, the change score was not statistically different from 0. Many of the individual barrier items followed this trend, with larger decrease in the early implementation stage (change score range: -0.04 to -0.39) and smaller change in the later period (change score range: -0.25 to 0.13). None of these changes were statistically different from zero, except: not enough buy-in from other unit staff; not enough buy-in from physician staff; not enough leadership from nurses; and insufficient autonomy/authority.

**Figure 2:**
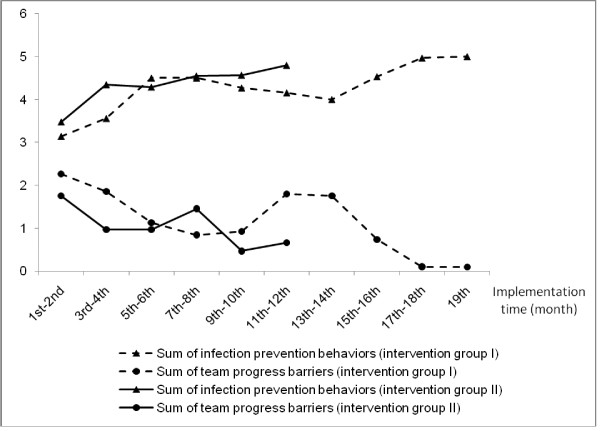
**Bimonthly numbers of perceived infection prevention behaviors and team progress barriers**.

The ability of team member reports to estimate infection prevention behaviors and progress barriers was demonstrated by the expected trends in improved perceived group infection prevention behaviors and fewer team progress barriers over time (Figure 2). Furthermore, similar trends were observed for the two intervention groups, even though intervention group II lagged intervention group I by seven months. The robustness of these findings provides additional validation of the responsiveness and stability properties of the TCT.

### Measure validity

#### Construct validity

Table 3 presents findings from the construct validity analyses. As hypothesized, we found that the sum of barriers perceived is negatively associated with the sum of infection prevention behaviors (Pearson r = -0.35, p < 0.001). The correlation of individual items with the sum of infection prevention behaviors ranged between -0.13 to -0.37 (all p < 0.001). The strongest correlation were for insufficient buy-in from other staff members (r = -0.37), other nursing staff (r = -0.36), and other physician staff (r = -0.34) and insufficient leadership support from nurses (r = -0.31). Among respondents reporting poor team function, insufficient participation was significantly negatively related to prevention behaviors (r = -0.19, p = 0.001). The other contributors to poor team function were not (p = 0.21 to 0.48).

**Table 3:** Construct validity: correlation of infection prevention activities with team progress barriers*

	Sum of Infection Prevention Activity Questions	
**Barrier Questions**	**Pearson correlation coefficient**	**p value**

Sum of #15a to #15 m	-0.350	< 0.001**
a. Insufficient knowledge	-0.205	< 0.001**
b. Lack of team member consensus	-0.249	< 0.001**
c. Not enough time	-0.262	< 0.001**
d. Lack of quality improvement skills	-0.242	< 0.001**
e. Not enough buy-in from other staff members	-0.374	< 0.001**
f. Not enough buy-in from other physician staff	-0.343	< 0.001**
g. Not enough buy-in from other nursing staff	-0.361	< 0.001**
h. Burden of data collection	-0.187	< 0.001**
i. Not enough leadership support from executives	-0.158	< 0.001**
j. Not enough leadership support from physicians	-0.290	< 0.001**
k. Not enough leadership support from nurses	-0.306	< 0.001**
l. Insufficient autonomy/authority	-0.271	< 0.001**
m. Inability of team members to work together	-0.130	< 0.001**
Sum of #15 m1 to #15m5	-0.046	0.412
m1. Insufficient participation	-0.191	0.001
m2. Some members do not value the others' contributions	-0.073	0.209
m3. Low or no feeling of being a team	-0.054	0.346
m4. Personality conflicts	0.041	0.475
m5. Poor conflict resolution skills	-0.068	0.237

*Individual-level data (N = 1,406) were used for these analyses; N = 322 for 15 m1 to 15m5 items as these are asked only if respondent indicates that the team could not work together more than one-half the time.

** p < 0.00384 (Bonferroni correction to account for multiple comparison was used.)

#### Convergent and discriminant validity

Convergent validity of the TCT barrier items were confirmed through a significant negative correlation with the TFS (r = -0.56, p = 0.007). Discriminant validity was demonstrated through a non-significant correlation with the overall PES score (r = -0.12, p = 0.68).

The correlation of the individual barrier items with their related convergent measure (TFS, various subscales) and discriminant measure (hospital staffing and resource adequacy subscale from the PES) is presented in Table 4. At the item level, expected relationships with TFS-specific subscales were generally confirmed, reflecting convergent validity. There were two items, insufficient knowledge of evidence and leadership support from physicians that did not demonstrate significant negative correlation with the hypothesized TFS subscale. However, the TFS scale on team autonomy appears to be associated with the barrier items on buy-in from other staff (r = -0.59, p = 0.004) and other nursing staff (r = -0.57, p = 0.006) in the unit, although we did not initially hypothesize an association between these measures. As hypothesized, we did not find significant correlation of the PES subscale on hospital staffing and resource adequacy with any barrier items.

**Table 4:** Convergent and discriminant validity: correlation with Team Functioning Survey and Practice Environmental Scale

Barrier items	Convergent Measure TFS subscale (n = 22 ICUs)	r	Discriminant Measure PES subscale (n = 15 ICUs)	r
Insufficient knowledge of evidence	Team self-assessed skill	-0.20	Staffing/resource adequacy	-0.08
Lack of team consensus	Participation and goal agreement	-0.61**	Staffing/resource adequacy	-0.15
Not enough time	NA		Staffing/resource adequacy	0.27
Lack of quality improvement skills	Team self-assessed skills	-0.52*	Staffing/resource adequacy	-0.11
Not enough buy-in from other staff	Team autonomy╪	-0.59**	Staffing/resource adequacy	-0.21
Not enough buy-in from other physician staff	NA		Staffing/resource adequacy	0.08
Not enough buy-in from other nursing staff	Team Autonomy╪	-0.57**	Staffing/resource adequacy	-0.16
Burden of data collection	NA		Staffing/resource adequacy	-0.02
Not enough leadership support from executives	Organizational support	-0.52*	Staffing/resource adequacy	-0.33
Not enough leadership support from physicians	Organizational support	-0.34	Staffing/resource adequacy	0.02
Not enough leadership support from nurses	Organizational support	-0.43*	Staffing/resource adequacy	0.05
Insufficient autonomy/authority	Team autonomy	-0.61**	Staffing/resource adequacy	-0.23
Inability of team members to work together	Participation and goal agreement	-0.45*	Staffing/resource adequacy	0.23

* p < 0.05; **p < 0.01; ╪ most strongly significant correlation observed, not initially hypothesized; NA indicates no prior hypothesis regarding relationship

#### Predictive validity

We predicted that the fewer perceived barriers would be associated with a shorter time to desired outcomes. However, our findings did not support this hypothesis (Table 5). There were small and non-significant relationships between the sum of barriers with the time to first quarter with no CLABSI and the time to first quarter when the unit staff consistently performed the five prevention behaviors. It is possible that low variance in these outcomes may have limited our ability to detect these associations. Of the 46 ICUs participating in the trial, all achieved zero CLABSI (at some point during the collaborative) and 25 achieved consistent performance in all five prevention behaviors during the intervention period. Twenty-six (57%) were able to achieve zero CLABSI by month one, with another seven achieving this goal by month two (15%). Average CLABSI for Intervention group I fell from 4.71 per thousand line days in the first month to 0.27 in the fourth month. Similarly, average CLABSI rate for group II was 5.60 per thousand line days in the first month of the implementation but only 0.12 by the fourth month. There may also be multiple influences on these outcomes, among which team perceived barriers may play a relatively modest role.

**Table 5:** Predictive validity: association of TCT barrier questions to infection prevention behaviors and CLABSI

	Time to 1st Quarter with No CLABSI (n = 22 ICUs)		Time to 1st Quarter with All 5 Prevention Behaviors (n = 15 ICUs)	
	
Barrier questions	**Coef**.	P value	**Coef**.	P value
Sum of #15a to #15 m (Scores in the first month)	-0.019	0.802	-0.020	0.822
a. Insufficient knowledge	0.060	0.801	0.215	0.376
b. Lack of team member consensus	-0.074	0.807	0.000	0.999
c. Not enough time	0.090	0.696	-0.475	0.232
d. Lack of quality improvement skills	-0.254	0.468	0.050	0.855
e. Not enough buy-in from other staff members	-0.044	0.861	-0.004	0.986
f. Not enough buy-in from other physician staff	-0.070	0.707	-0.305	0.183
g. Not enough buy-in from other nursing staff	-0.057	0.830	-0.075	0.784
h. Burden of data collection	-0.204	0.477	0.064	0.827
i. Not enough leadership support from executives	-0.176	0.493	0.089	0.787
j. Not enough leadership support from physicians	-0.007	0.975	-0.186	0.480
k. Not enough leadership support from nurses	-0.010	0.968	-0.500	0.220
l. Insufficient autonomy/authority	-0.285	0.336	-0.115	0.726
m. Inability of team members to work together	-0.166	0.684	-0.333	0.564

*Based on unadjusted Cox proportional hazard regression models.

## Discussion

Implementation success for healthcare quality and safety interventions can vary significantly across teams. Assessing differences in team context and progress can help QI team members make adjustments over the course of the intervention and help researchers design more effective interventions. In addition, identifying factors associated with successful teams can increase the likelihood of implementation success for future teams. However, measures used for these assessments must be reliable, valid, and responsive in order to be useful for these purposes.

In this study, we examined the measurement properties of the TCT, a short instrument that has been used to track implementation progress for an intervention to reduce bloodstream infections within ICUs [13]. TCT measures evaluated in this study included participation in intervention components, perceptions of unit performance on infection prevention behavior, and key barriers to team progress including lack of leadership support and physician engagement, which are often central to implementation success [6,19-21]. Our study found support for the temporal stability, construct validity, and responsiveness of QI team member reports on intervention activities, perceived unit-level behaviors, and barriers to team progress from this instrument. At both the item and aggregate level, the TCT measures we evaluated were responsive during periods of high implementation activity and stable during periods of low implementation activity. Furthermore, the general trajectories in behaviors and barriers were as predicted, with greater number of prevention behaviors and fewer barriers observed over the course of the intervention. The greatest changes took place within the first six months of the intervention. These findings attest to the value of these measures for detecting change and tracking the course of implementation progress.

Construct validity for the evaluated measures was generally good. A hypothesized overall relationship between infection prevention behaviors and barriers was supported by a significant moderate correlation. Individual barrier items also demonstrated significant associations, with items related to buy-in from physician, nursing, and other clinical staff being the strongest. This finding appears reasonable given that improved ICU behavior depends on the commitment of the entire ICU staff, not just of the QI team members.

Convergent and discriminant validity results were also as hypothesized. The TFS [7], which assesses aspects of team effectiveness, provides a good overall match to the TCT barrier items and the expected association is supported by a significant and moderately strong correlation. Similarly, moderate to strong correlations were observed for TCT items to corresponding TFS subscales. Discriminant validity is also good, with the TCT barrier items demonstrating weak and non-significant association with the overall and staffing/resource subscale of the PES, a more general measure of nursing work environment. Together, these findings indicate that the TCT barrier items provide valid measures of team effectiveness and functioning.

However, barriers to team progress at baseline did not predict time to zero CLABSI rate as hypothesized. Constraint in the variance of this outcome may have limited our ability to detect an association between our TCT measures and the key clinical outcome of interest in the intervention. In addition, while barriers on the QI team very likely affect intervention outcomes, they may not be the strongest influences. Although these results did not confirm our original hypothesis regarding predictive validity, most findings from this study support the reliability, validity, and responsiveness of the TCT measures we examined. Furthermore, the low burden of this instrument, requiring less than 10 minutes to complete and reporting only once a month, contributes to the instrument's feasibility for use in busy clinical settings such as ICUs.

Demonstration of a practical, valid, and responsive measure of context is important given the growing interest in better theoretical and practical understanding of how context affects implementation success of effective QI interventions [5,7,22,23]. Among the contextual elements that may influence QI success are staffing, work environment, safety climate, teamwork, implementation activities, organizational culture, learning and mindfulness, leadership support, and engagement of participants. The TCT emphasizes key internal processes and group psychosocial traits of the QI team. Although there is little consensus around how to define and measure implementation context, team functioning and effectiveness have been examined in a number of QI studies [1]. Unfortunately, few studies of team effectiveness have used validated instruments.

Additional work to more clearly define and validate measures of implementation context will be important for advancing the research in this area. Several instruments, although not specific to healthcare, have been validated. Anderson and West developed and validated the factor structure, internal consistency, predictive validity, and within-team consensus for the Team Climate Inventory, which focused on the climate for innovation within work groups [11]. Wheelan and Hochberger developed and validated the Group Development Questionnaire, a general measure of the trajectory of group formation and development [24]. These instruments are clearly valuable to the understanding of team-based interventions. However, the generalized concepts, while useful for research purposes, may be less relevant for QI team members seeking opportunities to achieve success in implementing a specific intervention. Furthermore, these instruments are often longer and, therefore, less feasible to complete on a routine basis in busy clinical settings. Finally, the concepts they measure may not reflect the rapid changes that take place within a QI intervention. Teams and collaborative faculty often need more frequent feedback on the effects of changes they have made to improve care.

Measures of implementation context specific to QI collaboratives have been developed and are being validated, including a 14-item instrument by Dückers *et al*. [25] measuring team organization, internal support (leadership and organization), and external support (external change agents). The TFS provides a measure of team functioning with demonstrated reliability that has been used to assess team effectiveness in QI collaborative [7]. Our study contributes to this literature on team context measures for QI efforts by demonstrating the responsiveness of the tool to changes in barriers to team progress and unit-level infection prevention behaviors over the course of the intervention. We also demonstrated the impact of team functioning on intervention effectiveness through the significant negative association between barriers to team progress and unit-level behaviors in infection behaviors. Finally, our study demonstrated reliability for TCT through good temporal stability during a stable phase of the intervention period. These are critical properties to assess when tracking progress of a team-based intervention.

Our study has several limitations. First, not all items included in the TCT were evaluated as part of this study. Validity of items related to the number of times the teams met with executives and hospital boards, nursing turnover, and diffusion practices are challenging to assess for the measurement properties of interest, because they represent events that may not consistently take place from month to month and gold standard data for validation are not readily available. Second, many items included in this instrument-such as the elements of CUSP, educational activities, and infection prevention behaviors-are specific to the interests and needs of the target intervention. This will limit the applicability of this measure and our findings to other QI interventions. However, there are also benefits of this approach to assessing implementation context. Specifically, tailoring measures to QI intervention can make the assessments more relevant and useful for teams participating in a specific intervention. For example, team members can discuss specific barriers with each other and senior leaders and resolve issues that hamper implementation progress. As we expect the diffusion of the bloodstream infection prevention intervention to expand, the findings from this study should offer useful insights for future QI teams. Third, the lack of significance for several correlations with absolute r value between 0.21 and 0.33 that we observed in the discriminant validity analyses may be due to the small number of ICUs included. However, given that most of the correlations were weak, we expect that our overall conclusion would be robust even if a larger sample of ICUs had been available. Fourth, the findings of validity analyses that relied solely upon TCT data (*e.g*., the association of prevention behaviors and team barriers) may be artificially strengthened due to common method bias. Fifth, we did not first establish the convergence of member reports for the team level measures, because we relied on a conceptual basis for 'team' (*i.e*., assignment to team) rather than an empirically-driven one that requires substantial correlation of member reports. We reasoned that member perceptions cannot be assumed to converge because team members likely have different skills, roles, and experience and, further, may have different observations of team experience. Despite these differences, individual members are core contributors to and observers of the team. Therefore, their reports, in aggregate, provide valid reflections of team experience, irrespective of their level of convergence. Finally, as a longitudinal effectiveness project conducted under naturalistic conditions, data quality was a concern. Team-level response rate was challenging to determine because QI team composition can change over time. There was little assurance that member responses were not seen by others within or outside the QI team because conditions were not controlled by the research team. Many ICUs had months when TCT data were not submitted. This missing data led to the exclusion of some ICUs from temporal stability and responsiveness analyses. However, a comparison of the characteristics and CLABSI outcomes of included and excluded ICUs found no significant differences, suggesting that our conclusions regarding the psychometric properties of the TCT were sound.

## Conclusions

Overall, our study provides evidence of measurement reliability, validity, and responsiveness for a new tool that can assist researchers and practitioners in better understanding how context affects implementation success. By having validated measures on implementation context that are practical to administer longitudinally, researchers can more readily conduct rigorous studies on time varying contextual factors that affect implementation success, strengthening the evidence base on successful spread of efficacious team-based interventions. QI teams participating in an intervention should also find data from a validated tool to be more convincing and useful for identifying opportunities to improve implementation within their own teams. Given the variable success in QI interventions, the TCT offers a valid and feasible tool to help improve the probability of success and advance the science of QI.

## Competing interests

The authors declare that they have no competing interests.

## Authors' contributions

KSC participated in the design of the study, led the development and implementation of the analytic plan and data interpretation, and drafted the manuscript. YJH acquired the data, performed all statistical analysis, and participated in data interpretation and drafting of the paper. LHL participated in conceptualization and design of the study and provided critical review of earlier drafts. JAM conceived of the study, participated in its design and drafting the manuscript, and provided critical review of earlier drafts. All authors read and approved the final manuscript.

## Authors' information

KSC is an Associate Professor in the Department of Health Policy and Management at the Johns Hopkins Bloomberg School of Public Health. JAM is an Associate Professor in the same department and also has a joint appointment with the Department of Anesthesiology and Critical Care Medicine at Johns Hopkins School of Medicine. LHL is Assistant Professor with the Department of Anesthesiology and Critical Care Medicine at Johns Hopkins School of Medicine. YJH is currently a doctoral candidate in the Department of Health Policy and Management at the Johns Hopkins Bloomberg School of Public Health.

## Supplementary Material

Additional file 1 **The Team Check-up Tool**. A copy of the full Team Check-Up Tool instrument. Click here for file
