# Supplementary material for: Validity and usefulness of members reports of implementation progress in a quality improvement initiative: findings from the Team Check-up Tool (TCT)
Source: Implement Sci. 2011 Oct 3;6:115. doi: 10.1186/1748-5908-6-115 (PMC3205035; doi:10.1186/1748-5908-6-115)
Supplement: Additional file 1 — The Team Check-up Tool. A copy of the full Team Check-Up Tool instrument. [file 1748-5908-6-115-S1.DOC]

| **Table S1. The Team Check-up Tool** | | |
| --- | --- | --- |
| **ICUID: Month/year for which data provided:** | | |
| **Please answer the following questions with respect to the last month only:** | | |
| 1. Please indicate the CUSP activities in which your team participated by checking all that apply: | | □ AM Briefing □ Exec. Partnership  □ Shadowing □ Daily Goals  □ Learning from a Defect  □ Science of Safety video |
| 2. Which of the following did your team undertake on the unit to teach unit staff how to prevent bloodstream infections (check all that apply)? | | □ Internal seminar □ IC* visit/ talk  □ In-services/ demos  □ New written policy □Posted the steps  □ Put the protocols on all clipboards  *Infection Control |
| 3. What portion of staff on the unit consistently uses the following? | |  |
| a. Appropriate hand hygiene | | □ Few □ Some □ Most □ All |
| b. Chlorhexidine skin preparation | | □ Few □ Some □ Most □ All |
| c. Full-barrier precautions during line insertion (maintaining a sterile field) | | □ Few □ Some □ Most □ All |
| d. Subclavian vein placement | | □ Few □ Some □ Most □ All |
| e. Removing unnecessary lines | | □ Few □ Some □ Most □ All |
| 4. How often did your senior executive meet with your team regarding the ICU project (please enter number)? | | _______________ |
| 5. How often did your senior executive review your unit’s ICU performance data (please enter number)? | | _______________ |
| 6. Did your team have a chance to present your unit’s ICU performance data to senior hospital/health system leadership? | | □ No □ Yes |
| 7. Did your team have a chance to present your unit’s ICU performance data to the hospital/health system Board? | | □ No □ Yes |
| 8. How many times did your team meet (please enter number)? | | _______________ |
| 9. How often did your team review your performance data (please enter number)? | | _______________ |
| 10. How often did your team share your performance results broadly with ICU staff (please enter number)? | | _______________ |
| 11. If data were shared with ICU staff, please indicate how feedback was provided by checking all that apply: | | □ Verbal Report □ Poster  □ Written Report □ Other:___________ |
| 12.a. Did anyone on your quality improvement team permanently leave?  b. Check here if you ADDED anyone to the team. □ | | □ The hospital □ The unit □ The team |
| 13. Did your team or unit attempt to/ were you asked to share information or detailed advice about this project with…? | | □ Another ICU, same hospital; □ Non-ICU, same hospital; □ Outside hospital |
| 14. Has there been any non-routine event in your unit that has distracted staff from this work (*e.g.*, re-organization; death of staff; sentinel event; accreditation, etc.)? | | □ No □Yes, specify _______________ |
| **15. In the past month, did any of the following slow your team’s progress?** | | |
| a. Insufficient knowledge of evidence supporting interventions | □ Never/ rarely □ Under ½ the time □½ the time  □ Over ½ the time □ Almost always/Always | |
| b. Lack of team member consensus regarding goals | □ Never/ rarely □ Under ½ the time □½ the time  □ Over ½ the time □ Almost always/Always | |
| c. Not enough time | □ Never/ rarely □ Under ½ the time □½ the time  □ Over ½ the time □ Almost always/Always | |
| d. Lack of quality improvement skills | □ Never/ rarely □ Under ½ the time □½ the time  □ Over ½ the time □ Almost always/Always | |
| e. Not enough buy-in from other staff members in your area | □ Never/ rarely □ Under ½ the time □½ the time  □ Over ½ the time □ Almost always/Always | |
| f. Not enough buy-in from other physician staff in your area | □ Never/ rarely □ Under ½ the time □½ the time  □ Over ½ the time □ Almost always/Always | |
| g. Not enough buy-in from other nursing staff in your area | □ Never/ rarely □ Under ½ the time □½ the time  □ Over ½ the time □ Almost always/Always | |
| h. Burden of data collection | □ Never/ rarely □ Under ½ the time □½ the time  □ Over ½ the time □ Almost always/Always | |
| i. Not enough leadership support from executives | □ Never/ rarely □ Under ½ the time □½ the time  □ Over ½ the time □ Almost always/Always | |
| j. Not enough leadership support from physicians | □ Never/ rarely □ Under ½ the time □½ the time  □ Over ½ the time □ Almost always/Always | |
| k. Not enough leadership support from nurses | □ Never/ rarely □ Under ½ the time □½ the time  □ Over ½ the time □ Almost always/Always | |
| l. Insufficient autonomy/authority | □ Never/ rarely □ Under ½ the time □½ the time  □ Over ½ the time □ Almost always/Always | |
| m. Inability of team members to work together | □ Never/ rarely □ Under ½ the time □½ the time  □ Over ½ the time □ Almost always/Always | |
| **If response to 15m is ½ the time or more, did any of the following contribute:** | | |
| m.1. Insufficient participation of one or more team members | □ Never/ rarely □ Under ½ the time □½ the time  □ Over ½ the time □ Almost always/Always | |
| m.2. Some members do not value the contributions of other team members | □ Never/ rarely □ Under ½ the time □½ the time  □ Over ½ the time □ Almost always/Always | |
| m.3. Low or no feeling of being a team | □ Never/ rarely □ Under ½ the time □½ the time  □ Over ½ the time □ Almost always/Always | |
| m.4. Personality conflicts | □ Never/ rarely □ Under ½ the time □½ the time  □ Over ½ the time □ Almost always/Always | |
| m.5. Poor conflict resolution skills | □ Never/ rarely □ Under ½ the time □½ the time  □ Over ½ the time □ Almost always/Always | |
